# Mortality in an Italian nursing home during COVID-19 pandemic: correlation with gender, age, ADL, vitamin D supplementation, and limitations of the diagnostic tests

**DOI:** 10.18632/aging.202307

**Published:** 2020-12-22

**Authors:** Biagio Cangiano, Letizia Maria Fatti, Leila Danesi, Giacomo Gazzano, Marina Croci, Giovanni Vitale, Luisa Gilardini, Stefania Bonadonna, Iacopo Chiodini, Chiara Francesca Caparello, Antonio Conti, Luca Persani, Marco Stramba-Badiale, Marco Bonomi

**Affiliations:** 1 IRCCS Istituto Auxologico Italiano, Department of Endocrine and Metabolic Diseases, Milan, Italy; 2 IRCCS Istituto Auxologico Italiano, Laboratory of Endocrine and Metabolic Diseases, Cusano Milanino, Italy; 3 Department of Medical Biotechnology and Translational Medicine, University of Milan, Milan, Italy; 4 IRCCS Istituto Auxologico Italiano, Anatomic Pathology Unit, Milan, Italy; 5 IRCCS Istituto Auxologico Italiano, Laboratory of Geriatric and Oncologic Neuroendocrinology Research, Milan, Italy; 6 IRCCS Istituto Auxologico Italiano, Gastroenterology and Digestive Endoscopy, Milan, Italy; 7 IRCCS Istituto Auxologico Italiano, Department of Geriatrics and Cardiovascular Medicine, Milan, Italy

**Keywords:** hydroxychloroquine, serology, nasopharyngeal swab, sensitivity and specificity, activities of daily living, COVID-19

## Abstract

Introduction: The COVID-19 pandemic caused an increased mortality in nursing homes due to its quick spread and the age-related high lethality.

Results: We observed a two-month mortality of 40%, compared to 6.4% in the previous year. This increase was seen in both COVID-19 positive (43%) and negative (24%) residents, but 8 patients among those testing negative on the swab, tested positive on serological tests. Increased mortality was associated with male gender, older age, no previous vitamin D supplementation and worse “activities of daily living (ADL)” scores, such as Barthel index, Tinetti scale and S.OS.I.A. classification.

Conclusion: Our data confirms a higher geriatric mortality due to COVID-19. Negative residents also had higher mortality, which we suspect is secondary to preanalytical error and a low sensitivity of the swab test in poorly compliant subjects. Male gender, older age and low scores on ADL scales (probably due to immobility) are risk factors for COVID-19 related mortality. Finally, mortality was inversely associated with vitamin D supplementation.

Design: In this observational study, we described the two-month mortality among the 157 residents (age 60-100) of a nursing home after Sars-CoV-2 spreading, reporting the factors associated with the outcome. We also compared the diagnostic tests for Sars-CoV-2.

## INTRODUCTION

The year 2020 opened with, and has been forcefully defined by, the 2019 Coronavirus disease (COVID-19) global pandemic, which represents one of the most dramatic events of the last century, due to its severe impact in terms of public health effect and its long-term socio-economic implication. Starting form the first reported cases in China, in December 2019, a total of more than 50 million cases has already been confirmed in the first 11 months of 2020 with a total of 1,254,657 confirmed deaths in 219 different countries around the world [1] (https://www.who.int/emergencies/diseases/novel-coronavirus-2019). Italy was one of the most affected countries, and the North of the peninsula, especially Lombardy, was the most impacted area. A total of 935,104 confirmed cases of COVID-19 have been officially reported in the time between the very first reported case on the 21^st^ of February 2020, in the area of Milan, up until the 9^th^ of November 2020, and 41,394 deaths have been reported [2] (https://covid19.who.int/region/euro/country/it).

The incubation period for COVID-19 is thought to extend to 14 days, with a median interval of 4-5 days from exposure to symptom onset [3–5]. Presentations of COVID-19 have ranged from asymptomatic/mild symptoms to severe illness and mortality. In particular, in symptomatic patients, clinical presentation at illness onset may vary, although, over the course of the disease, most will experience fever, cough, difficulty breathing, fatigue, muscle or body aches, headache, new loss of taste or smell, sore throat, congestion or runny nose, nausea or vomiting, diarrhea [4, 6–11]. It was quite evident from the start of the pandemic that older adults, and those with chronic medical comorbidities such as male subjects, were particularly vulnerable and prone to a more severe outcome of the disease [4, 6–10].

Direct person-to-person transmission is the primary means of transmission of severe acute respiratory syndrome coronavirus 2 (SARS-CoV-2) [3]. It is thought to occur through close-range contact, mainly via direct inspiration of infected respiratory droplets or indirectly from touching an infected surface then touching one’s eyes, nose, or mouth.

Given their congregate nature and resident population (e.g., advanced age and frequent underlying chronic health conditions), nursing homes have been at high risk of being affected by COVID-19. Indeed, the effects of SARS-CoV-2 in nursing homes where the infection developed, have been devastating, accounting for a disproportionate, although not precisely estimated, number of deaths all over the world, including Italy [12–21].

Here we are presenting the data concerning the experience in a long-term care facility of Milan, where the COVID-19 disease heavily impacted the health of the resident population between March and April 2020. Our data aims to quantify the impact of SARS-CoV-2 on mortality in nursing homes and to point out the factors related to its severity as well as the limitations of diagnostic tests used to manage the spread of this infective disease.

## RESULTS

**Figure 1:**
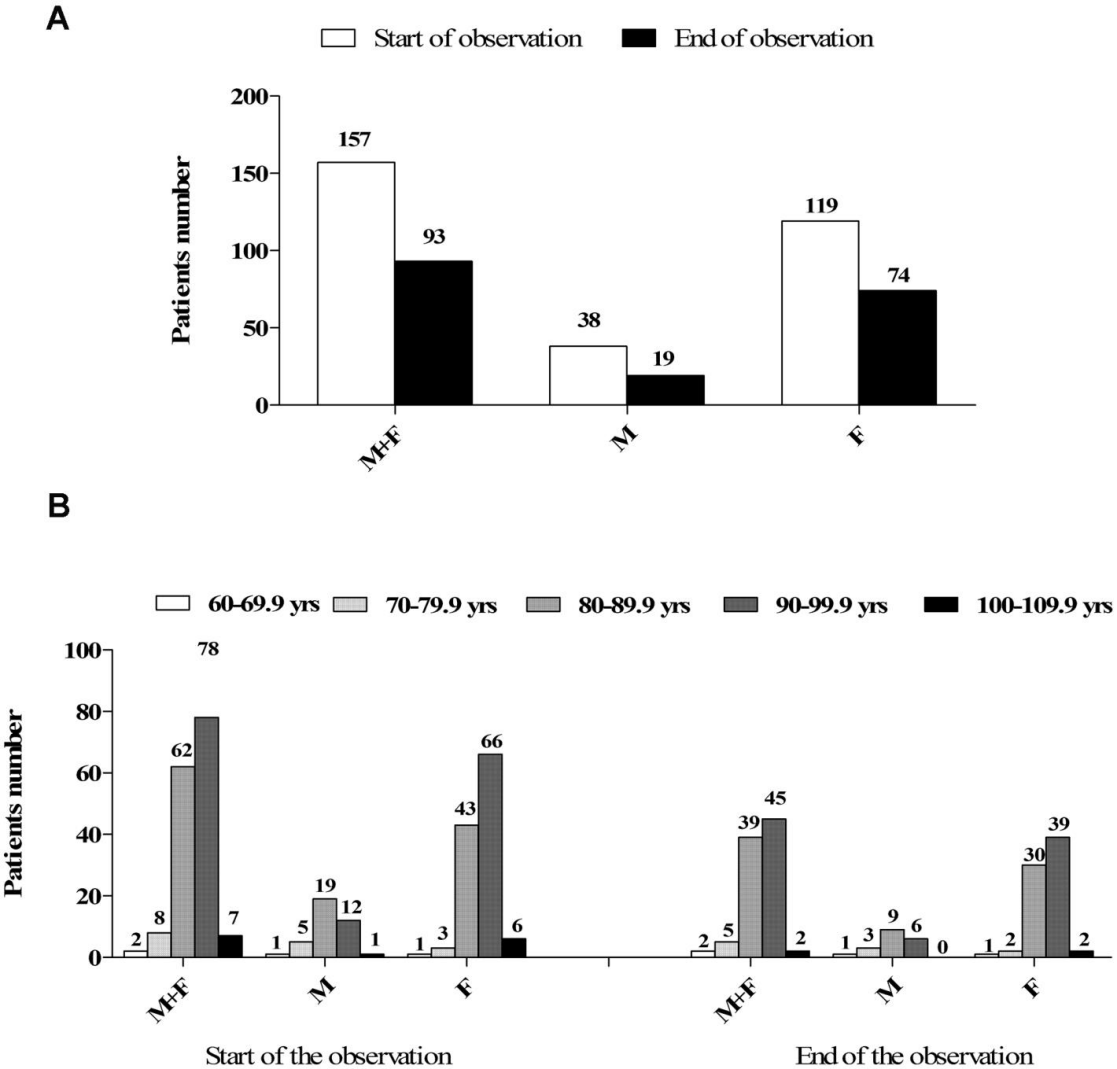
(**A**) Numerosity and gender distribution of the nursing home guests at the beginning and at the end of the observation study. (**B**) Distribution of the nursing home guests by gender and age decades either at the beginning and the end of the observation study.

A total of 157 subjects were hosted in the IRCCS Istituto Auxologico Italiano “Mons. G. Bicchierari” nursing home on March 1^st^, 2020, most of whom aged between 80 and 100 years old (Table 1 and Figure 1A). At the end of the two-months observational study, 93 subjects were still alive (Figure 1B). Characteristics of the cohort here studied are reported in Tables 1–3, Supplementary Table 1. 63% of subjects tested positive for SARS-CoV-2 on the nasopharyngeal swab test. 37% remained negative despite serial testing and were therefore isolated on a different floor of the building. In particular, 6 out of 11 subjects that showed symptoms and tested negative on their first viral RNA swab test, resulted positive on repeating the test, whilst the rest continued to test negative. Also, serological evaluation found 8 positive patients among the patients who were negative to the swab, still alive at the end of the observation and who performed the serological evaluation (n. 34), whereas it found 4 negative results among the 40 patients positive to nasopharyngeal swab who were alive at the end of the observation and accepted to perform the test for anti-COVID-19 immunoglobulins.

**Table 1:** Age and BMI of the nursing home guests.

**Parameter**	**Sub-categories**	**Sex**	**Mean**	**SD**	**Median**
***Age** (years)*	*All guests*	M+F	89.86	6.53	90.68
		M	87.3	7.27	87.74
		F	90.67	6.09	91.18
	*All deceased guests*	M+F	91.2	6.5	90.6
		M	88.8	6.7	88.7
		F	92.2	5.1	91.9
	*Deceased guests SARS-CoV-2 POS*	M	89.9	7.7	89.4
		F	91.8	6.4	91.3
	*Deceased guests SARS-CoV-2 NEG*	M	86.2	4.4	86
		F	92.7	3.9	93
***BMI** (Kg/m^2^)*	*All guests*	M+F	21.65	4.44	21,75
		M	22.93	4.11	22.2
		F	21.24	4.48	20.8

M: male; F: female.

**Table 2:** Co-morbidities of the nursing home guests.

**Subcategories /morbidity**	**≥ 1**	**None**	**R**	**CV**	**N**	**H**	**EM**	**GE**	**Other**
***Male + Female***	79.6%	20.4%	27.4%	66.2%	68.1%	9.5%	41.4%	24.2%	65.6%
***Male***	71.1%	28.9%	26.3%	65.7%	57.8%	15.7%	34.2%	15.7%	57.8%
***Female***	82.4%	17.6%	27.7%	66.4%	71.4%	7.5%	44.5%	26.9%	68%
***SARS-CoV-2 POS***	21.5%	78.5%	26.8%	73.1%	70.9%	9.6%	41.9%	29%	70.9%
***SARS-CoV-NEG***	11.1%	88,9%	29.6%	62.9%	68.5%	9.2%	46.2%	16.7%	62.9%

≥ 1:at least 1 associated co-morbidity: None: absence of co-morbidity; R: diseases of the respiratory system; CV: disease of the cardiovascular system; N: disease of the nervous system; H: disease of the hematopoietic system; EM: disease of the endocrine-metabolic system; GE: disease of the gastroenteric system; Other: any other disease of any other system.

**Table 3:** Chronic therapies of the nursing home guests.

**Subcategories /treatment**	**Any**	**ACEi**	**ARBs**	**GC**	**AC**	**AP**	**VD**
***Male + Female***	78.3%	22.9%	14%	8.2%	19.7%	28.6%	17.8%
***Male***	71%	15.7%	10.5%	10.5%	23.7%	23.7%	18.4%
***Female***	80.6%	25.2%	15.1%	7.5%	18.4%	30.2%	17.6%
***SARS-CoV-2 POS***	84.9%	25.8%	13.9%	8.6%	20.4%	30.1%	21.5%
***SARS-CoV-NEG***	75.9%	20.3%	16.7%	7.4%	20.3%	29.6%	12.9%

ACEi: Angiotensin-converting enzyme inhibitors; ARBs: Angiotensin II Receptor Blockers; GC: Glucocorticoids; AC: Anticoagulants; AP: Antiplatelets; VD: Cholecalciferol.

During the two months of observation in the nursing home, we observed a mortality of 40.1 % (63 deaths/157 patients), whereas in the same two months of the previous year the mortality was 6.4% (10 deaths/155 patients). Among COVID-19 positive residents (to any nasopharyngeal swab), mortality rose to43%, whilst among COVID-19 negative residents, mortality rose to 24%.

A comparison between the clinical characteristics of patients who passed away and those who survived is reported in Table 4. Patients who survived showed younger age, normal BMI, better ADL scores, and chronic treatment with cholecalciferol compared with patients who passed away.

**Table 4:** Comparison of different variables in SARS-CoV-2 positive guests.

**Parameter**	**Deceased (42)**	**Surviving (56)**	***p-value***
Males	13	15	*0.7*
hydroxychloroquine	5	28	***<0.0001***
Heparin	13	31	***0.02***
O_2_	17	18	*0.4*
ACEi	7	17	*0.2*
ARB	5	8	*0.8*
antiplatelet	9	19	*0.3*
anticoagulants	7	12	*0.6*
glucocorticoids	3	5	*1*
Vitamin D	3	17	***0.005***
Respiratory diseases	7	18	*0.1*
Diabetes	6	5	*0.5*
Hypertension	16	32	*0.07*
Age	91.3±6.7	88.8±6.1	***0.05***
BMI	21.0±4.6	23.2±4.7	***0.02***
Barthel Score	7.1±9.0	23.6±22.8	***<0.0001***
S.OS.I.A. C	1.3±0.5	1.7±0.5	***0.0002***
S.OS.I.A. M	1.0±0.2	1.2±0.4	***0.004***
S.OS.I.A. S	1.0±0.2	1.1±0.3	*0.3*
Tinetti Index	2.2±3.4	8.6±7.9	***0.0007***

Data are shown as absolute number or mean±SD or absolute number with range or percentage in parentheses, respectively.

Logistic regression analyses for factors associated with mortality found that a higher age (p=0.03), male gender (p=0.03), positivity to SARS-CoV-2 (p=0.002), and lower scores to Barthel (p=0.003), Tinetti (p=0.001) and S.OS.I.A (in this case only for the sub-scale C, p=0.004) scales were significantly associated with a poor outcome.

The same associations, with the exception of male gender (p=0.3) and obviously positivity to the swab, were confirmed when evaluating only COVID-19 positive patients.

When evaluating chronic concurrent therapies, we found a statistically significant inverse correlation of vitamin D (cholecalciferol) treatment with mortality (p=0.05). To assess if cholecalciferol chronic treatment was independently associated with a better outcome, we then performed a logistic regression in COVID-19 positive subjects evaluating together all the above mentioned factors significantly associated with mortality (age, gender, ADL scores, BMI and cholecalciferol chronic treatment), confirming the protective role of vitamin D supplementation (p=0.04).

Finally, regression analyses studying COVID-19 specific therapy (O_2_, hydroxychloroquine, heparin) found only an inverse relation of COVID-19 mortality with hydroxychloroquine therapy (p=0.03)

## DISCUSSION

During the initial spreading, the COVID-19 pandemic had an exponential expansion and a high death toll in the first regions to be hit by the new virus, such as Lombardy, especially in frail subsets of patients as the older residents of nursing homes. This is not surprising considering the challenges faced in nursing homes in previous infectious outbreaks and epidemics, such as the Severe Acute Respiratory Syndrome (SARS) epidemic in 2003 [22]. Indeed, nursing homes have been documented as having a high risk of transmission rates for infectious diseases for several reasons, including sharing common areas, facilities, sources of air, food, water and health care in a crowded institutional background. Furthermore, free movements of visitors, staff and residents allowed, until the first Ministerial and Regional restriction laws at the beginning of March, a continuous transmission with the external community. Moreover, it has been documented that COVID-19 outbreaks are related to facilities’ size, with larger facilities (>150 bed) as the “Mons. G. Bicchierai” nursing home, being those at greater risk of spreading of the virus [23]. This could explain why around 2/3 of our subjects tested positive for SARS-CoV-2.

With the aim of containing as much as possible the spread of the virus in long-term care homes, several governmental agencies and professional bodies have released guidance for nursing homes during the COVID-19 pandemic (WHO, 2020 https://apps.who.int/iris/bitstream/handle/10665/331508/WHO-2019-nCoV-IPC_long_term_care-2020.1-eng.pdf; CDC, 2020 https://www.cdc.gov/coronavirus/2019-ncov/healthcare-facilities/prevent-spread-in-long-term-care-facilities.html; British Geriatric Society, 2020 https://www.bgs.org.uk/resources/covid-19-managing-the-covid-19-pandemic-in-care-homes).

Nevertheless, SARS-CoV-2 had a large spreading among frail subjects, and recent literature reports mortality in subjects aged between 80 and 89 years to be 54.3% (60% and 48% in males and females respectively), and 52.3% in subjects aged above 90 years [24]. The mean age of our patients was 89 (Table 1) years and the mortality in the two months following the spreading of the virus was only slightly less than the one reported in literature despite the efforts of the healthcare professionals.

Comparing the age of death of the patients of our nursing home with the data coming from the general population (data from ISTAT, https://www.istat.it/it/archivio/212512), it is higher than the national mean (82.6, 80.3, 84.9 years for both sexes, male and female, respectively), as expected among residents of nursing homes, who are commonly old and frail patients.

The comparison with the previous year mortality of the same months in the same nursing home showed a 6.7 times increased mortality, clearly related to the epidemic of COVID-19. The abovementioned increase was highest in subjects testing positive for SARS-CoV-2, but apparently, also among the patients testing negative, there was a higher mortality compared to the previous year. Besides the fragility of all the patients due to geriatric syndromes, this increased mortality could be due to different reasons. On one hand, we should take into account that patients struggled with the privation of the visits of their relatives due to the isolation caused by the virus, and the stop to the physical therapy and social activities of the nursing home. Many non-autonomous subjects with dementia or impairment in ADL, showed a decline of their functional cognitive performances during this period. All these factors can lead to refusal of food and immobility in bed, with the inexorable worsening of previous psychophysical conditions [25].

On the other hand, the increased mortality in the SARS-CoV-2 negative residents could be due to a low sensitivity of the tests to detect SARS-CoV-2 RNA, therefore including among the negatives some affected patients. In fact, eleven symptomatic subjects testing negative to the first nasopharyngeal swab, immediately repeated the examination and six were found to be positive, thus showing a low real-life sensitivity, probably due to difficulty in the sample collection. The procedure to collect samples using nasopharyngeal swab, despite being performed by trained professionals, could be particularly problematic and imprecise, especially in older subjects with impaired mobility of the neck and low compliance to the procedure. We cannot exclude that other patients with a negative result were not falsely negative indeed. To support this hypothesis, we found 8 subjects with a positive serology for SARS-CoV-2 who were negative on any previous nasopharyngeal swab. However, as a post-mortem examination has not been performed in any of these patients, we cannot provide a clear explanation for the increased mortality in COVID-19 negative subjects.

If we assume that the sensitivity (97.9 %) and specificity (98.5 %) of serological evaluation are very high, real-life clinical swab sensitivity in this subset of patients appears to be 81.8% (36 true positives, 8 false negatives), probably due to the abovementioned sample collection problems as already reported in literature [26]. This value could also be overestimated since we checked the positivity using serological evaluation of anti SARS-CoV-2 immunoglobulins only in living patients at the end of the observation: the prevalence of false negatives among the subjects who died could be higher if COVID-19 was the cause of some of these deaths.

What is even more challenging to explain are the seemingly 4 false positive results to the nasopharyngeal swab. In fact, this discordance is less clear since SARS-CoV-2 RNA detection should be highly specific and is hardly explainable the 86.6% specificity (26 true negatives, 4 false positives) on the basis of the abovementioned serologic tests. Nonetheless, the nasopharyngeal swab of these 4 residents was only mildly positive at the evaluation using Xpert® Xpress SARS-CoV-2 kit (see patients and methods), thus we can presume that the viral load was quite low and maybe not enough to stimulate an immune reaction with a resulting serological conversion [27]. Furthermore, a possible prevalent involvement of other immune responses, such as native and different adaptive immunity activation should also be considered [28].

The data from our nursing home confirm a higher mortality to be related to older age and male gender, even considering only patients above 60 years old. Moreover, a lesser degree of independence measured with each one of Barthel’s score, Tinetti scale, or C and M scales of S.OS.I.A., is strongly related to a poor outcome in this subset of patients and explains the high COVID-19 mortality in nursing homes. We don’t know if this could be related to hypercoagulability in immobilized patients as suggested by some authors in other populations [29–33]. A low BMI and no heparin treatment in COVID-19 positive patients were also associated with increased mortality but both were not confirmed in logistic regressions. On the other hand, in our cohort, differently to what was reported in other published cohorts [7, 8, 10, 18, 34–42], it was not possible to establish a clear relationship between mortality of COVID-19 affected residents and the presence of any of the associated co-morbidities reported in Table 2. Nonetheless, it is worth noting that around 80% of the residents had at least one associated comorbidity (Table 2). The reverse relation of mortality with chronic treatment with cholecalciferol has already been reported in the literature [43] and we confirm this association also in the subjects above 60 years old; a recent quasi-experimental study in 66 residents of a French nursing home, showed that vitamin D3 supplementation (oral bolus of 80,000 IU) was associated with less severe COVID-19 infection and a higher survival rate [44].

It is known that vitamin D acts as an immune modulator, but it is not known how this affects the COVID-19 disease. Nonetheless, considering the impact of the new virus in older subjects, the commonly prevailing deficiency in 25OH vitamin D in this subgroup, and the absence of contraindications to this supplementation, the administration of cholecalciferol in older subjects could be strongly advocated. On the contrary the inverse association of mortality with hydroxychloroquine therapy found in our study could be affected by a severe selection bias. Hydroxychloroquine was prescribed only in patients with better ECG tracings and those receiving less drugs that might induce QT interval prolongation, such as antipsychotic and antidepressant agents, thus being probably fitter then those who did not receive this therapy. In order to understand the effects of these drugs on COVID-19, it is therefore important to look at RCTs, which are still ongoing [45].

Concluding, we report the factors associated with increased mortality in non-autonomous COVID-19 positive subjects aged between 60 and 100 years, with the privileged point of view of a nursing home suddenly affected with the new SARS-CoV-2 virus. In this paper we provide the real-life sensitivity of validated viral detection analyses, performed by trained operators, in partially compliant non-auto sufficient patients. This information is important for public health policymaking since, according to our data, all residents in these facilities should be considered as vulnerable and more likely to spread infection both within the premises and externally. It is important to separate residents that test positive from those who test negative. Negative results may be falsely so, and therefore it is also equally important to manage the latter as being potentially positive in order to avoid further spread of the virus, potentially also to healthcare professionals., Our data further contributes to the body of evidence pointing out a possible protective role of cholecalciferol supplementation during the COVID-19 pandemic, with a statistically significant inverse correlation of vitamin D (cholecalciferol) treatment with mortality in nursing home residents. Finally, we also report the usefulness of ADL scores to predict a poor outcome in older patients affected with COVID.

## MATERIALS AND METHODS

In this one-center observational study, we evaluated the data of 157 subjects, residents at the nursing home of “Mons. G. Bicchierai 1-2” of IRCCS Istituto Auxologico Italiano between March and April 2020. This facility is home to residents aged between 60 and 100 years, suffering from various disabilities, and requiring assisted living, medical care, and rehabilitation services.

For each patient, the age, the BMI at the beginning of the evaluation, the presence of cardiovascular, neurological, endocrinologic, haematologic and gastroenterological comorbidities, diabetes and hypertension were collected. The concomitant chronic treatment with ACE inhibitors (ACEi), angiotensin receptor blockers (ARBs), oral anticoagulants, antiplatelet, cholecalciferol, glucocorticoids was also reported; in particular, subjects undergoing cholecalciferol treatment were all treated with a two-times-a-month 25000 UI regimen. Patients were hospitalized in three different floors of the nursing home, and the floor hosting each patient was also recorded.

## Activities of Daily Living (ADL) scores

Different scores investigating the patients’ degree of independence in the Activities of Daily Living (ADL) were also considered:

1- The Barthel index, developed in the late 1950s by the English nurse Barthel, measures the degree of a patient’s independence and is composed of 10 items that investigate common ADL. Each item is assigned a score, the sum of which (maximum 100) indicates the degree of autonomy of the patient in carrying out daily life activities [46].

2- The Tinetti scale (or Performance Oriented Mobility Assessment) assigns a score to the person's ability to maintain balance while performing tasks related to ADL. The Tinetti scale is organized in two distinct parts: one concerns the balance and is made up of 9 items with a score that can vary from 0 to a maximum of 16; the other analyzes the gait and consists of 7 items with a score between 0 and 12 [47].

3- The S.OS.I.A. classification (Which means assistance intermediate observation form) is used to evaluate the degree of fragility of nursing home patients in Lombardy, with a score ranging from 1 to 8. This classification is the result of 3 specific and dichotomous variables / indicators: the indicator M for mobility aimed to ADL; the indicator C for cognitive ability / behavior; the indicator S for the severity of comorbidities (cardiac pathology, hypertension, vascular pathologies and the blood and lymphatic system, respiratory, ENT, gastrointestinal, hepatic, renal, genital-urinary, musculoskeletal cutaneous, neurological, endocrine-metabolic, psychiatric-behavioral) [48].

Deaths occurring during the period of the evaluation were also reported.

## Treatment for COVID-19

In patients testing positive for SARS-CoV-2, the treatment specifically aimed to treat COVID-19 was also recorded, including newly introduced anticoagulants (subcutaneous Enoxaparin sodium 4000 IU), hydroxychloroquine (200mg twice a day for 7 days), and newly introduced O_2_ therapy. It has to be noted that hydroxychloroquine was prescribed only in subjects showing a normal corrected QT interval at electrocardiography, and without any interfering drug. All treated patients underwent a second ECG during therapy and in case of QT interval prolongation the drug was withdrawn. All patients necessitating invasive or non-invasive ventilation systems (13 patients in the two months period) were admitted to intensive care units (ICU), and provided with the appropriate therapy.

## COVID-19 diagnostic tests

When the epidemic started, we looked for viral RNA among the 157 patients of the nursing home. The test was repeated twice and then every month or in case of suspected symptoms. Positivity to SARS-CoV-2 was assessed using PCR for viral detection using nasopharyngeal swab specimens (all collected by two trained operators).

For the initial evaluation of positivity, viral RNA was detected using Xpert® Xpress SARS-CoV-2 kit from Cepheid, a real-time reverse transcription PCR targeting two genes: N2–nucleocapsid gene and E–envelope protein gene; limit of detection 10 copies/μL.

No potential unintended cross reactivity with other organisms (Human coronavirus 229E, Human coronavirus OC43, Human coronavirus HKU1, Human coronavirus NL63, SARS-coronavirus, MERS-coronavirus, Bat coronavirus, Adenovirus e.g. C1 Ad. 71, Human Metapneumovirus hMPV, Parainfluenza virus 1-4, Influenza A, Influenza B, Influenza C, Enterovirus e.g. EV68, Respiratory syncytial virus Rhinovirus, Chlamydia pneumoniae Haemophilus influenzae, Legionella pneumophila, Mycobacterium tuberculosis, Streptococcus pneumoniae, Streptococcus pyogenes, Bordetella pertussis, Mycoplasma pneumoniae, Pneumocystis Jirovecii PJP, Parechovirus, Candida albicans, Corynebacterium diphtheriae, Legionella non-pneumophila, Bacillus anthracis Anthrax, Moraxella catarrhalis, Neisseria Elongata and Meningitidis, Pseudomonas aeruginosa, Staphylococcus epidermidis, Staphylococcus Salivarius, Leptospira, Chlamydia Psittaci, Coxiella Burnetii Q-Fever, Staphylococcus Aureus) is expected based on the in silico analysis.

Particular feature of this test is that it has three possible outcomes: positivity if both genes are detected, negativity if no gene is amplified, and mild positivity whenever only one of the two genes is found to be positive.

Subsequent tests for viral RNA were performed using iAMP® SARS-CoV-2 Detection Kit from Atila BioSystem, a real-time reverse transcription isothermal amplification test, performed directly from raw samples. Limit of Detection is 20 copies/μL. No cross-reactivity with other organisms (Human Coronavirus 229E, Human coronavirus OC43 strain ATCC VR-759, Human coronavirus HKU1, human coronavirus NL63, SARS coronavirus B093, MERS coronavirus isolate NL140422, Human metapneumovirus isolate 00-1, Human parainfluenza virus 1 NM001, Human parainfluenza virus 2 isolate VIROAF10, Human parainfluenza virus 3 strain HPIV3/AUS/3/2007) is expected due to *in silico* Cross-reactivity analysis.

Moreover, a serological test was performed in patients alive and willing to participate one month after the end of the observation. To detect IgG anti Sars-CoV2the “CLIA” assay *LIAISON® SARS-CoV-2* *S1/S2* was used. Clinical sensitivity after 15 days from the infection is 97.9% (89.1 – 99.6%), and clinical specificity is 98.5% (95% CI: 97.5 – 99.2%). The limit of detection is 3.8 AU/mL (https://www.diasorin.com/sites/default/files/allegati_prodotti/liaison_sars-cov-2_s1_s2_igg_0.pdf).

## Statistical methods

Statistical analysis was performed using SPSS, version 21.0, statistical package (SPSS Inc., Chicago, IL, USA). We used Mann-Whitney and Fisher exact tests to compare all possible risk factors between COVID-19 positive deceased subjects and COVID-19 positive patients alive at the end of the observation. Logistic regression analyses were used to evaluate independent factors associated with mortality, at first in the whole cohort, and then studying only subjects testing positive for SARS-CoV-2 to nasopharyngeal swab. In these analyses we evaluated: positivity for SARS-CoV-2 to nasopharyngeal swab, age, gender, BMI, hypertension, diabetes mellitus, measures of ADL, and in which floor of the building the patient was hospitalized. To evaluate ADL the same analysis was repeated three times, each one with a different scale (Barthel, Tinetti, and S.OS.I.A).

A similar analysis was performed in positive subjects taking into account previous therapies with ACE inhibitors (ACEi), angiotensin receptor blockers (ARB), oral anticoagulants, antiplatelet, cholecalciferol, and glucocorticoids; finally, in positive patients the association of mortality with treatments against COVID-19 was also evaluated.

## Ethic approval

Compliant to all relevant ethical standards.

## Supplementary Material

Supplementary Table 1
