# Supplementary material for: Mortality in an Italian nursing home during COVID-19 pandemic: correlation with gender, age, ADL, vitamin D supplementation, and limitations of the diagnostic tests
Source: Aging (Albany NY). 2020 Dec 22;12(24):24522–34. doi: 10.18632/aging.202307 (PMC7803543; doi:10.18632/aging.202307)
Supplement: Supplementary Table 1 [file aging-12-202307-s001.pdf]

SUPPLEMENTARY TABLE

Supplementary Table 1. Activity of Daily Living (ADL) scores of the nursing home guests.

Parameter	Sex	Mean	SD	Median
Barthel score (0-100)	M+F	15.15	20.38	5
	M	16.44	21.71	5
	F	14.73	20	5
Tinetti score (0-28)	M+F	5.66	7.29	1
	M	5.97	7.53	1
	F	5.57	7.24	1
S.OS.I.A. score (1-8)	M+F	2.47	1.98	1
	M	2.52	1.72	3
	F	2.43	2.06	1

M: male; F: female.
